# Perceiving Natural Speed in Natural Movies

**DOI:** 10.1177/2041669519860544

**Published:** 2019-07-19

**Authors:** Mikako Kobayashi, Isamu Motoyoshi

**Affiliations:** The University of Tokyo, Tokyo, Japan

**Keywords:** motion, biological motion, naturalness, event perception

## Abstract

The visual system uses the physical laws of nature as constraints for perceiving objects and events. Images violating natural laws would therefore tend to be perceived as unnatural. To understand vision’s implicit knowledge of natural speed in the real world, we examined visual tolerance to artificial speed deviations in 22 natural movies. For most movies, perception could tolerate deviations from original speed by as much as a factor 2×. However, for movies including human body movements or falling objects, perception only tolerated a significantly narrower range of speed deviations. In general, human observers are poor at judging the naturalness of speed in natural scenes except for events involving gravitational or biological motions.

## Introduction

Objects and events in nature are governed by various physical laws that give rise to specific regularities in audiovisual information reaching perceptual systems. The human brain presumably uses these natural laws as constraints for inferring external objects and movements from sensory information (e.g., Marr, 1982). Dynamic events are determined by various physical and biological laws such as inertia, gravity, friction, and so on. These laws are thought to be reflected in the sensory information about dynamic events and to be used in perceiving the characteristics of dynamic events. If the visual system potentially “knows” some of the natural laws and statistical structures inherent in sensory information, images deviating from natural laws might then be perceived as “unnatural.”

It is known that observers easily perceive static images as unnatural if light–dark polarities are reversed (Anstis, 2005) or luminance-color correlation deviates from the original (Nakano et al., 2009). For dynamic events, humans can judge the naturalness in simple physical movements of a geometric object relatively accurately (e.g., Ceccarelli et al., 2018; Twardy & Bingham, 2002). However, these findings do not necessarily indicate that we can judge the naturalness of complex real-world events such as the flow of a water stream or the waving of trees in the wind. To address this issue, we examined whether observers can readily discriminate and reliably report movie naturalness/unnaturalness for a variety of natural movies played at variable speeds.

## Methods

**Figure 1.**
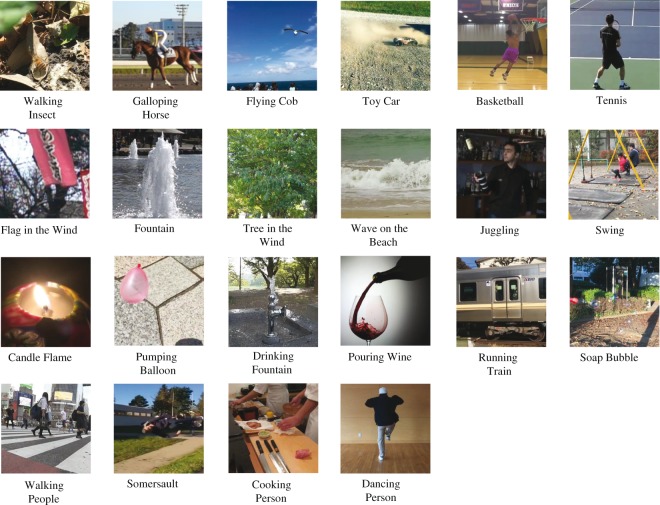
Snapshots of natural movies used in the experiment.

Visual stimuli consisted of 22 natural movies (3.4 × 3.4 deg). Snapshots of movies are shown in Figure 1. Thirteen of the movies were taken by a high-speed video camera (240 or 960 fps), and the other nine movies were downloaded from the Internet. Visual stimuli were displayed on a gamma-corrected LCD monitor with a refresh rate of 60 Hz. The experiment was approved by the research ethics committee at The University of Tokyo and consent forms were completed.

On each trial, a 1,060-ms section was randomly chosen from the original movie and presented in the center of a dark background. To control play speed, an appropriate number of frames were skipped from the original movie. For example, a 240-fps movie in which three out of every four frames are skipped will be played on the 60-Hz display at the same speed as the dynamic event in reality.

Ten observers determined the fastest and the slowest speeds at which the scene in the movie was perceived as “natural” using a method of adjustment. Fastest and slowest speed judgments were examined in different blocks.

The adjustment values were factors of 1.125 or 1.25 for increases, and 0.875 or 0.75 for decreases. These values were decided by our preliminary observation. For example, to measure the slower limit, observers increased play speed if the movie appeared unnaturally slow and decreased play speed if the movie appeared natural. Observers repeated this procedure and pressed a button to decide when play speed straddled the perceptual border between natural and unnatural. In each block (fastest and slowest) judgments were conducted three times for each movie.

## Results

**Figure 2.**
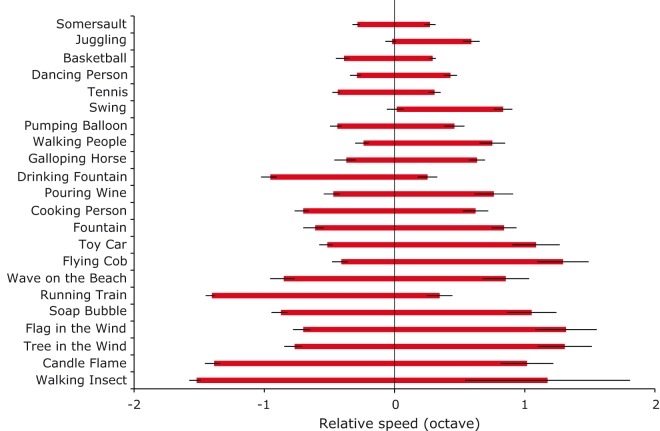
The range of perceived natural speeds for each scene. Error bars indicate ±1 *SE* across observers.

Figure 2 shows the range of perceived natural speeds for each scene relative to its physical natural speed. The range of natural speeds for most scenes is very wide, but the range for scenes including human body movement or falling of objects (Somersault, Juggling, Basketball, Dancing Person, Tennis, Swing, Pumping Balloon, Walking People, and Cooking Person) was narrower than those obtained for the other scenes, *t*(*14*) = 5.40, *p* < .001, Welch’s unequal variances *t*-test. The range of perceived natural speeds varies greatly depending on scene type and observers display limited sensitivity to speed deviations in most scenes except for scenes involving human movement or falling objects.

## Discussion

The present results suggest that observers, while unable to offer consistent judgments of natural speed for most natural scenes, can apply knowledge of “naturalness” to human body movements and free-fall events. While data were collected for a limited number of scenes, we offer the following potential interpretations and implications.

Observers may have a relatively higher sensitivity to the naturalness of human-body movement speed because of mechanisms involved specifically in processing biological motion (Johansson, 1973). Indeed, the ability to detect and recognize various properties such as actions, sexuality, and emotion from human biological motion (e.g., Troje, 2002) using limited cues of human body movements might enable observers to perceive subtle speed changes and unnaturalness.

While observers are able to detect unnatural speeds for free-falling objects for different gravity constants (Twardy & Bingham, 2002), observers are not always sensitive to the natural speeds of falling movements. For example, we found that the range of perceived natural speed for waterfall movements (e.g., fountains) is broad. This may be because different speeds could elicit different but plausible perceptual interpretations. Because the motion of liquids affects viscosity perception (Kawabe, Maruya, Fleming, & Nishida, 2015), observers might interpret a change in the physical speed of a flowing fountain as a change in water viscosity. Likewise, an artificial speed change for fire or for trees waving in the wind could be compatible with changes in natural physical factors such as wind strength.

## References

[bibr1-2041669519860544] Anstis S. (2005). Homage to Peter Thompson: The Tony Blair illusion. Perception, 34, 1417–1420. doi:10.1068/p5398 16355745 10.1068/p5398

[bibr2-2041669519860544] Ceccarelli F. La Scaleia B. Russo M. Cesqui B. Gravano S. Mezzetti M. Zago M. (2018). Rolling motion along an incline: Visual sensitivity to the relation between acceleration and slope. Frontiers in Neuroscience, 12, 406. doi:10.3389/fnins.2018.00406 29988401 10.3389/fnins.2018.00406 PMC6023988

[bibr3-2041669519860544] Johansson G. (1973). Visual perception of biological motion and a model for its analysis. Perception & Psychophysics, 14, 201–211. doi:10.3758/BF03212378

[bibr4-2041669519860544] Kawabe T. Maruya K. Fleming R. W. Nishida S. (2015). Seeing liquids from visual motion. Vision Research, 109, 125–138. doi:10.1016/j.visres.2014.07.003 25102388 10.1016/j.visres.2014.07.003

[bibr5-2041669519860544] Marr D. (1982). Vision. San Francisco, CA: W. H. Freeman.

[bibr6-2041669519860544] Nakano L. Takeuchi T. Motoyoshi I. Li Y. Adelson E. Nishida S. (2009). The effect of color saturation and luminance contrast on color naturalness [Abstract]. Journal of Vision, 9, 1040. doi:10.1167/9.8.1040

[bibr7-2041669519860544] Troje N. F. (2002). Decomposing biological motion: A framework for analysis and synthesis of human gait patterns. Journal of Vision, 2, 371–387. doi:10.1167/2.5.2 12678652 10.1167/2.5.2

[bibr8-2041669519860544] Twardy C. R. Bingham G. P. (2002). Causation, causal perception, and conservation laws. Perception & Psychophysics, 64, 956–968. doi:10.3758/BF03196799 12269302 10.3758/bf03196799

